# Interplay between organic solvent geometry and divalent cation dynamics in divalent metal batteries†

**DOI:** 10.1039/d5ra00757g

**Published:** 2025-04-07

**Authors:** Nazifa Jahan Pranti, Sharifa Faraezi, Tomonori Ohba, Argyrios V. Karatrantos, Md Sharif Khan

**Affiliations:** a Center for Interdisciplinary Chemistry Research (CICR) Dhaka Bangladesh sharifkhanjnu@gmail.com; b Graduate School of Science, Chiba University 1-33 Yayoi, Inage Chiba 263-8522 Japan; c Luxemburg Institute of Science and Technology 5 Avenue des Hauts-Fourneaux L-4362 Esch-sur-Alzette Luxembourg

## Abstract

This study investigates the interplay between organic solvent geometry and divalent cation dynamics in liquid electrolytes, emphasizing their relevance for energy storage systems. Using classical molecular dynamics simulations, the structural and transport properties of Mg^2+^ and Ca^2+^ were evaluated in cyclic (ethylene carbonate, EC; propylene carbonate, PC) and linear (ethyl methyl carbonate, EMC) solvents in the presence of TFSI^−^ anions across a range of temperatures. The results reveal that Mg^2+^ exhibits superior diffusion compared to Ca^2+^ due to its smaller ionic radius and weaker ion–pair interactions. Diffusion increases with temperature, following the solvent trend EC > EMC > PC. Coordination analysis showed compact solvation shells for both cations, with Ca^2+^ forming denser structures and demonstrating higher residence times compared to Mg^2+^. Solvent geometry significantly influenced solvation dynamics, with cyclic solvents enhancing ion coordination and linear solvents reducing solvation due to steric hindrance. These findings underscore the critical role of solvent structure and ion dynamics in optimizing divalent-ion battery performance, positioning Mg^2+^ as a promising candidate for sustainable energy storage solutions.

## Abstract 2

Elucidating the local structure and the dynamic nature of divalent cations under different solvent geometries.

## Graphical abstract

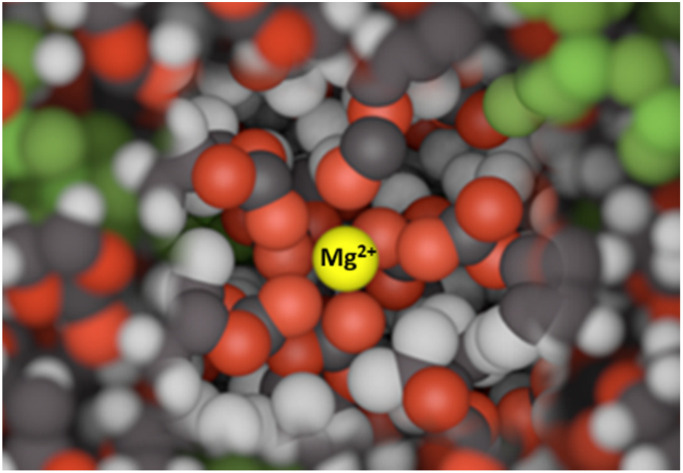

## 1. Introduction

The growing demand for portable electronic devices and electric vehicles, and increasing concerns about climate change have driven the development of sustainable energy storage technologies.^1,2^ Renewable energy sources are being utilized to alter fossil fuels, addressing these concerns by generating energy without producing greenhouse gas emissions.^3^ Rechargeable Li-ion batteries (LIBs) dominate the global power market due to their superior energy density and extensive technological and industrial development.^4–6^ However, adopting LIBs as a long-term energy storage solution will be hindered by issues with the limited natural abundance of lithium (only 0.002%), cost, and safety.^7^ Hence, to achieve a sustainable energy shift away from LIBs, different metal ion candidates such as Na^+^, K^+^, Mg^2+^, Ca^2+^, Zn^2+^, Al^3+^*etc.* are required^8–13^ to address the challenges and outperform existing energy density measurements in the metal ion batteries. As promising alternatives, low-cost, highly abundant divalent Mg^2+^ (2.15%)^14^ and Ca^2+^(4.86%)^15^ facilitate improved energy density compared to LIBs due to possessing two transferable electrons.^16^ Mg^2+^ exhibits a low electrode potential of −2.37 V (*vs.* SHE),^17^ including high specific capacitance (2205 mA h g^−1^) and high volumetric capacity (3833 mA h cm^−3^). In contrast, Ca^2+^ (ref. 18) has a volumetric capacity of 2052.6 mA h cm^−3^ and a redox potential of −2.87 V (*vs.* SHE) close to Li^+^. Another challenge conventional monovalent batteries (Li, Na, and K) encounter is the formation of detrimental needle-like dendrites on the metal anode interface^19^ during electrochemical plating/stripping processes. These dendrites can penetrate the separator, leading to short circuits and reduced battery performance. In contrast, Zhao *et al.* determined that smooth deposition occurs in 0.3 M all-phenyl-complex electrolytes where the Mg^2+^ effectively suppresses dendrite formation, which is less prominent than Li^+^ by adding an additive.^20–22^ Ca^2+^ does not form dendrite under typical battery conditions as readily as Li^+^ ion batteries. Calcium's larger ionic radius and distinct electrochemical characteristics reduce the risk of dendrite formation.^23^ Thus, there is increasing interest in using Mg^2+^ and Ca^+2^ as potential candidates for divalent-ion batteries, namely magnesium-ion batteries (MIBs) and calcium-ion batteries (CIBs).^24–26^

Electrolytes are one of the crucial elements in energy storage systems; therefore, numerous types of electrolytes have been reported in the literature, each distinguished by identical physical and chemical properties.^27–32^ Liquid electrolytes have been widely used in commercial energy storage systems due to their high ionic conductivity, thermal and chemical stability, and wide electrochemical stability window.^33–35^ However, the selection of suitable liquid electrolytes is critical to be compatible with magnesium and calcium anodes while maintaining safety, stability, and a wide operating potential window.^31,36^ The electrolyte phase serves to transport positively charged ions between the cathode and anode,^37,38^ the diffusion of the respective ions and their drift velocity in the electric field become extremely important to the battery's performance.^39^ Ion–solvent coordinating states in liquid electrolytes have been studied experimentally *via* Raman^36,40,41^ and infrared spectroscopy (IR).^42^ Pulsed-field gradient nuclear magnetic resonance (PFG-NMR) is utilized to determine the diffusion coefficients of ions and solvents within electrolytes, providing insights into ion (cations, anions) mobility, solvent behavior, and overall electrolyte performance in metal ion batteries.^43–46^ These properties are critical for optimizing electrolyte performance in energy storage systems and ensuring operational reliability under various conditions. While ^7^Li is a suitable nucleus for PFG-NMR diffusion measurements, the available isotopes of post-Li cations (such as Na or divalent) often lack a nuclear spin or suffer from short spin relaxation times, which prevent the application of spin echoes to observe the transport over the required time scales of at least several *ms*. Thus, despite many experimental efforts, understanding ion dynamics at the atomic level as well as the distinct local geometrical structure of solvents requires computational approaches, *e.g.*, the contribution of density functional theory (DFT) and classical molecular dynamics (MD) simulations aims to offer insight observation.^47–51^

Mg electrolytes based on the Mg(BH_4_)_2_ salt containing longer-(shorter-)chain solvents named THF and the glycol dimethyl ether (G1–G4) showed the lowest (highest) diffusion of Mg^+2^ and agglomeration rates.^52^ As a liquid electrolyte anion, perchlorate (ClO_4_^−^), hexafluorophosphate (PF_6_^−^) and tetrafluoroborate (BF_4_^−^) are feasible to react with Mg^2+^ ions, however, resulting in irreversible electrodeposition of Mg^2+^ limited their utilization.^53^ In such cases, bis(trifluoromethanesulfonyl)imide (TFSI) has been replaced because of its larger size and delocalized electrons, which leads to better dissociation and exhibits a high ionic conductivity. Lapidus *et al.*^54^ and Terada *et al.*^55^ reported the weak ion–pair interactions between the large TFSI^−^ anions and Mg^2+^ (ref. 56) in glycol dimethyl ether (G2 and G4), although poor solubility revealed in THF^57^ and acetonitrile (AN).^58^

Mg^2+^and Ca^2+^ stripping/plating of MIBs and CIBs are studied widely. However, challenges still exist, particularly in terms of selecting efficient liquid electrolytes with stability.

Seki *et al.* analyzed the interactions of various divalent metal cations (Ca^2+^ and Mg^2+^) with PC and TFSI^−^ anions by *ab initio* molecular orbital calculations. It was demonstrated that Mg^2+^ formed a more stable complex with PC and the TFSI^−^ anions than Ca^2+^. Thus, the diffusion coefficients of PC and TFSI^−^ were lower for Mg^2+^ than for the Ca^2+^ cation.^59^ To the best of our knowledge, there is not any experimental or simulation research investigating the diffusion coefficient of such electrolytes in EC and EMC solvents.

Further research is still needed to understand the Mg^2+^ and Ca^2+^ behavior in liquid electrolytes to further improve energy storage systems. Specifically their solvation shell, ion–pair interaction, the coordination number of ions and solvents, and structural effect of solvent in overall bulk and interface need to be evaluated thoroughly. In this study, we performed molecular dynamics simulations of cyclic (EC, PC) and linear (EMC) solvent-based liquid electrolytes of Mg^2+^, Ca^+2^, and TFSI^−^ at a range of temperatures from 303 K to 333 K. The local structure of these electrolytes has been revealed by considering the atomic-level pair correlation function. Furthermore, the diffusion mechanism and its dependence on the geometry of organic solvents were studied as a function of temperature.

## 2. Simulation procedures

All-atom molecular dynamics simulations were performed using the large-scale atomic/molecular massively parallel (LAMMPS) simulator MD code.^60^ Non-polarizable point-charge potential parameters for ethylene carbonate (EC), ethyl methyl carbonate (EMC), propylene carbonate (PC), and bis(trifluoromethanesulfonyl)imide (TFSI^−^) were adopted from a previous study and included in the Table S1,† (ref. 61 and 62) while the parameters for Mg^2+^ and Ca^2+^ were obtained from.^63^ Since non-polarizable force fields do not explicitly take into account the electronic polarization, we implement charge-scaling of ions (Mg^2+^, Ca^2+^, and TFSI^−^) by the factor of 0.7. Long-range coulombic interactions were calculated using the particle–particle particle–mesh (PPPM) method^64^ with a real-space cutoff of 1.2 nm. The van der Waals interactions were truncated at 1.0 nm, and the Lennard-Jones interactions were combined using Lorentz–Berthelot mixing rules.

Simulations were conducted in the NPT and NVT ensembles with time steps of 0.5 fs and 1.0 fs, respectively. Temperature and pressure control were maintained using the Nosé–Hoover thermostat and barostat, respectively. Simulation cells of 5 × 5 × 5 nm^3^ were constructed, containing the appropriate amounts of organic solvents (EC, EMC, and PC), cations (Mg^2+^ and Ca^2+^), and TFSI^−^ anions corresponding to a 0.5 M cationic and 1 M anionic concentration. The systems were annealed from 100 K to 700 K at 1 atm for 20 ns in the NPT ensemble. After equilibrium reached at 700 K, the temperature was gradually decreased in increments of 10 K.

During the cooling process, electrolyte configurations were sampled between 303 K and 343 K and used as initial states for 30 ns of NVT simulations, followed by 10 ns NPT simulations at each temperature. For the final analysis, trajectories from the last 10 ns of the production run were collected. The simulated bulk density values at 303 K were 1.33, 1.29, and 1.04 g cm^−3^ for EC, PC, and EMC solvents respectively, while the corresponding experimental values at this temperature ate 1.32, 1.20, and 1.006 g cm^−3^. The slightly higher simulated density values of the electrolyte systems compared to the experimental solvent densities can be attributed to the presence of cations and anions, as shown in Fig. S1.† The concentration of the simulated systems were determined after NPT equilibration, yielding cationic concentrations of 0.48 (±0.02), 0.47 (±0.002), and 0.48 (±0.002) M for EC, PC, and EMC, respectively.

## 3. Results and discussion

### 3.1 Diffusion

**Fig. 1:**
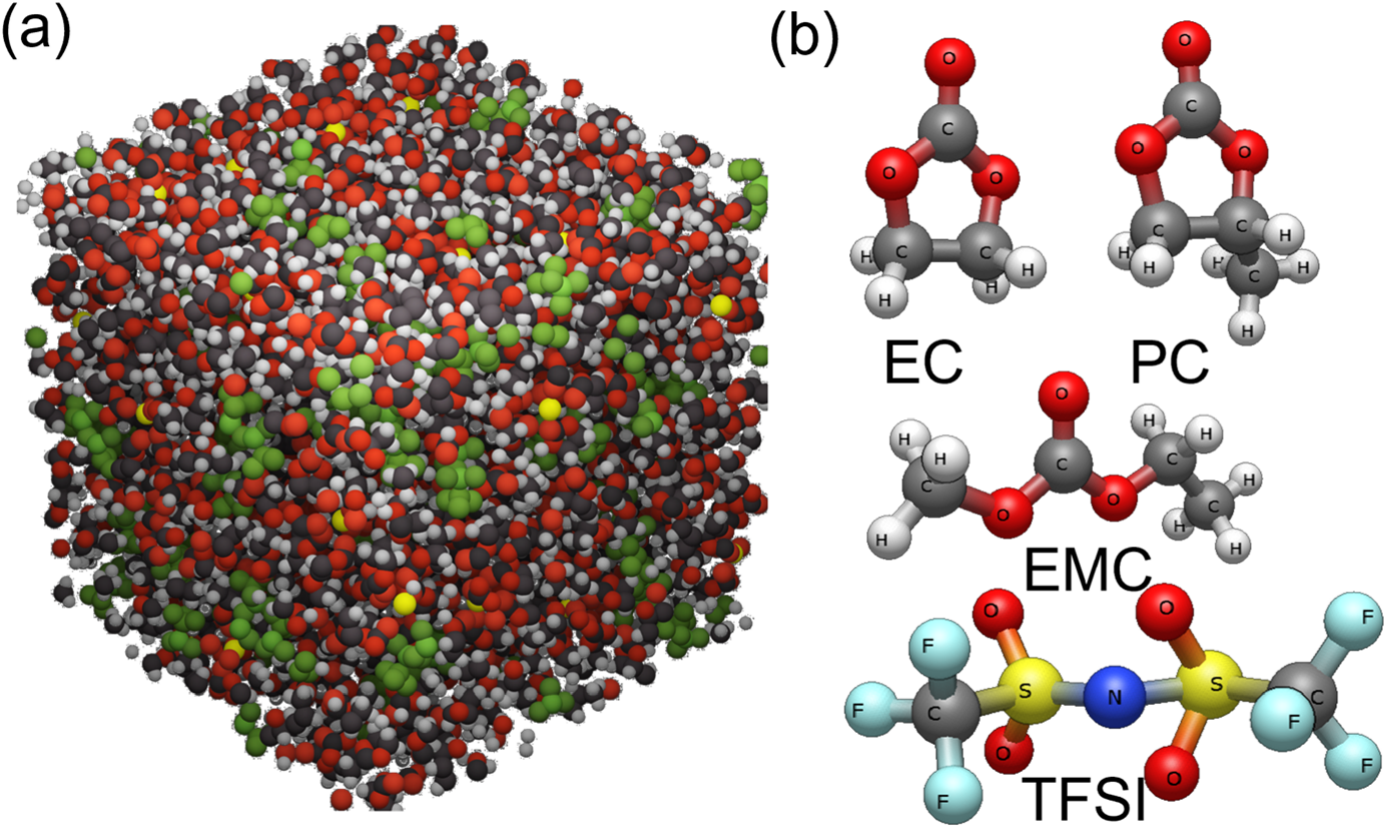
(a) Typical simulation system and the (b) ball and stick structure of the organic solvent and anion used in the simulations.

The representative simulation system employed in this study is depicted in Fig. 1(a). The electrolyte composition was determined to achieve a 1 M anionic concentration. The ball-and-stick representations of the solvents and the TFSI^−^ anion are presented in Fig. 1(b). The solvents include EC and PC, which possess cyclic geometries, and EMC, characterized by a linear geometry. These solvents exhibit distinct dielectric constants, reported as 88, 64.9, and 2.93 for EC, PC, and EMC, respectively.^65,66^ The TFSI^−^ anion, with its linear-like structure, plays a crucial role in modulating ion–pair interactions, demonstrating behavior distinct from conventional spherical anions.

It is known that the non-polarizable force fields do not explicitly take into account the electronic polarization.^67^ One way to remedy this issue is to incorporate electronic polarization in a mean-field way *via* charge rescaling.^68^ Leontyev and Stuchebrukhov proposed an electronic continuum theory (Molecular Dynamics in Electronic Continuum)^69^ to account for the effects of electronic polarization in non-polarizable force fields. In particular, their theory claims that ions in solutions should have charges scaled by about 0.7.^70^ In addition, for divalent ionic liquids in water or acetonitrile, quantum chemical calculations have shown that the charge scaling values should be well below the charge scaling factor of 0.8 typically used for monovalent ionic liquids. It was found that a value of 0.7 can give better MD density predictions in comparison to experimental data.^71^ Thus, the charges of Mg^2+^, Ca^2+^, and TFSI^−^ were scaled by the factor of 0.7 in order to mimic the experimental charge transfer in our non-polarizable MD simulations.

The diffusion coefficient was calculated from the simulated mean squared displacement (MSD) by using the following equation:^45^1
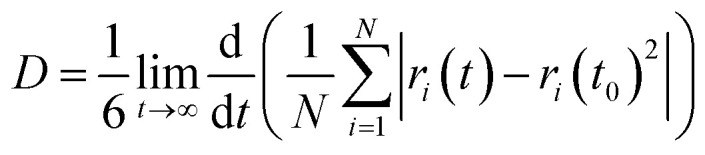
where (|*r*_*i*_(*t*) − *r*_*i*_(*t*_0_)|^2^) is the mean square displacement of an ion or molecule and is fitted to the linear diffusive regime.

**Fig. 2:**
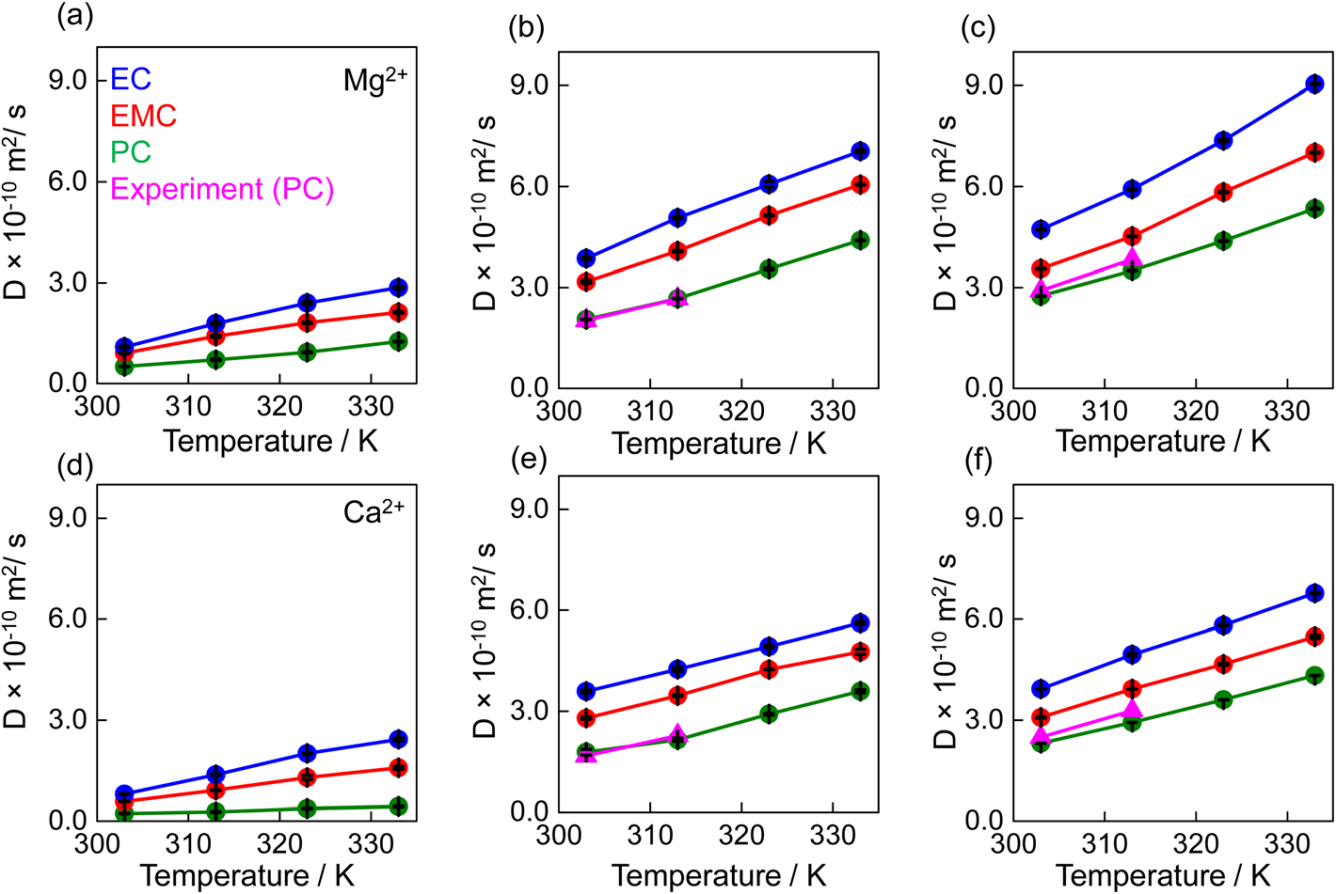
Simulated diffusion coefficients of Mg^2+^ ions (a), TFSI^−^ ions (b), and solvents (c) in Mg(TFSI)_2_ solutions. Similarly, simulated diffusion coefficients of Ca^2+^ ions (d), TFSI^−^ ions (e), and solvents (f) in Ca(TFSI)_2_ as a function of temperatures. The experimental diffusion co-efficient for TFSI^−^ and PC solvents are in a triangle shape with a pink color. Different solvents, EC, EMC and PC, are indicated by blue, red, and green colors, respectively.

The temperature-dependent simulated diffusion coefficients of Mg^2+^, Ca^2+^, TFSI^−^, and the various solvents are presented in Fig. 2(a–f) as a function of temperature. The diffusion coefficients for all components increase with temperature. First, the simulated diffusion coefficients of TFSI^−^ and PC solvent compare well with the experimental measurements.^45^ The solvents exhibit higher diffusion coefficients for both Mg^2+^ and Ca^2+^ systems than the anions and cations, while anions show higher diffusion than cations across all temperatures. Notably, diffusion increases by nearly threefold as the temperature rises from 303 K to 333 K. The choice of solvent significantly impacts the diffusion behavior of Mg^2+^, Ca^2+^, and TFSI^−^ following the trend EC > EMC > PC. The diffusion activation energy of Mg^2+^ and Ca^2+^ in different solvents was determined using Arrhenius fitting of the diffusion coefficients. The activation energy for Mg^2+^ diffusion was found to be 0.013 eV, 0.016 eV, and 0.014 eV in EC, PC, and EMC, respectively, while for Ca^2+^, the corresponding values were 0.011 eV, 0.014 eV, and 0.013 eV.

Despite its higher dielectric constant, PC demonstrates lower cation diffusion, attributed to the additional methyl group, which introduces bulkiness, steric hindrance, and favorable cation–anion interactions disrupting cation mobility.^72,73^ Comparing Mg^2+^ and Ca^2+^, Mg^2+^ consistently exhibits higher diffusion than Ca^2+^, regardless of the solvent or temperature it is in agreement to a recent study by Karatrantos *et al.*^45^ showing the same behavior by both experiments and simulations, which is in contrary to the experimental measurements by Seki *et al.*^59^ Unlike monovalent cations, divalent cations show an inverse relationship between ionic size and diffusivity, with smaller cations diffusing more rapidly; this has also been found in the experimental measurements.^29,45^ This variation in diffusion behavior of cation, anion and solvents is closely linked to the nature of the ion–solvent and cation–anion interactions and the local structures formed by the ions–solvents and in the electrolyte. Therefore in the later section of the manuscript we have quantify the pair correlations, coordination number and residence time to illustrate their direct impact on diffusion trends.

### 3.2 Local structure and coordination

The radial distribution function (RDF) is calculated to investigate the atomic level correlation between different pairs. Typically, the *g*(*r*) of two particles, such as A and B can be calculated as:^62^2
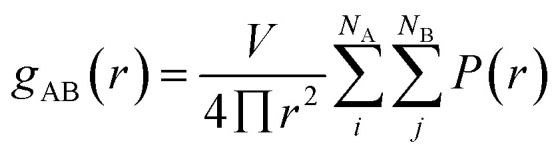
where *V* is the volume of the simulation system, *N*_A_ and *N*_B_ are the number of atoms A and B, respectively, and the probability of finding a B atom at a defined distance *r* from an A atom is denoted by *P*(*r*).

**Fig. 3:**
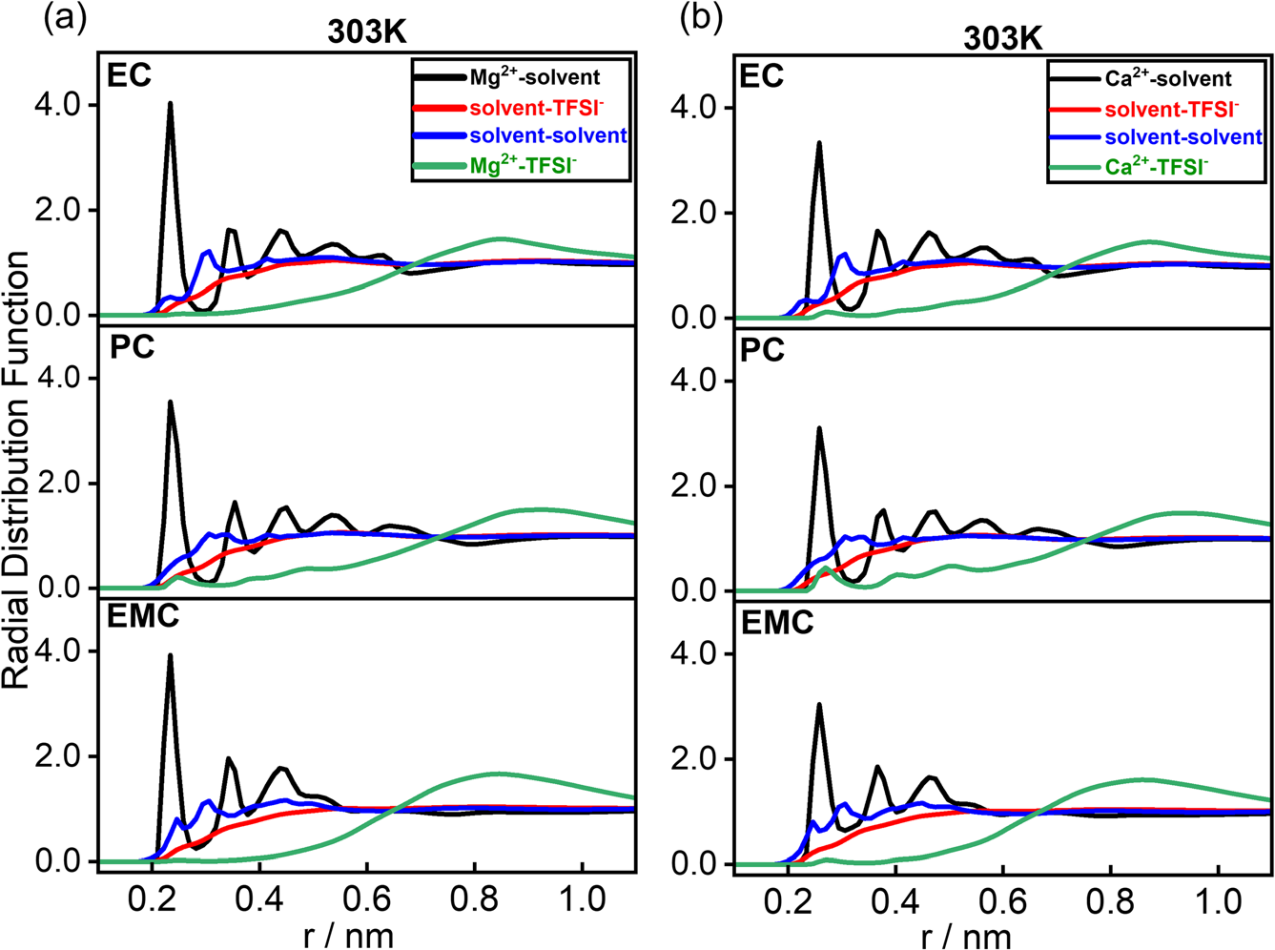
Radial distribution function of the different correlations in different solvents at 303 K temperature for Mg^2+^ (a) and Ca^2+^ (b) ion system.

The simulated radial distribution functions (RDFs) for various correlations are illustrated in Fig. 3(a and b). The solvent–solvent, Mg^2+^–solvent, TFSI^−^–solvent, and Mg^2+^–TFSI^−^ pairs are defined as follows: solvent–solvent interactions refer to the pairing between the carbonyl oxygen atoms of two solvent molecules; Mg^2+^–solvent interactions involve the coordination between Mg^2+^ and the carbonyl oxygen and other possible sites of the solvent; TFSI^−^–solvent interactions correspond to the pairing between the nitrogen of TFSI^−^ and the carbonyl oxygen of the solvent; and Mg^2+^–TFSI^−^ interactions represent the association between Mg^2+^ and the nitrogen of TFSI^−^. Both Mg^2+^ and Ca^2+^ exhibit dominant coordination with the solvents. The first solvation shell peaks for Mg^2+^ with EC, EMC, and PC appear at 0.23 nm, while those for Ca^2+^ appear at 0.26 nm, reflecting that both of the cations are forming strong solvation structure with all of the different solvents. However, the longer first neighboring distance for Ca^2+^-solvent is associated with its larger ionic radius. The layering structure in the cation–solvent RDFs which is observed in Fig. 3 (black lines) means that the cations are solvated strongly by the solvent, mainly due to the strong electrostatics between the divalent cations and the carbonyl oxygen (which has a negative charge) of the solvent. For solvent–solvent correlations, the first peaks for EC–EC, PC–PC, and EMC–EMC occur at 0.23 nm, 0.31 nm, and 0.25 nm, respectively. PC exhibits reduced accessibility to other PC molecules compared to EC and EMC. Notably, these solvent–solvent correlations remain unchanged when transitioning from Mg^2+^ to Ca^2+^. In contrast, TFSI^−^ does not exhibit sharp peaks with the solvents, showing a broad peak at approximately 0.55 nm across all solvent and cationic systems. There is a depletion layer of TFSI^−^ around the solvent up to that distance (0.55 nm) from the solvent (red lines in Fig. 3). In a previous work on divalent cations in PC solutions^45^ it was also observed a negligible correlation between that the Mg^+2^ and TFSI^−^ anions. For TFSI^−^–Mg^2+^ interactions, the first peaks appear at 0.85 nm, 0.25 nm, and 0.85 nm in EC, PC, and EMC, respectively. In the case of TFSI^−^–Ca^2+^, a pronounced peak is observed at 0.27 nm for all solvents, indicating stronger ion–pair interactions with Ca^2+^ compared to Mg^2+^. This trend is consistent across EC, EMC, and PC, confirming that Ca^2+^ forms stronger ion pairs with TFSI^−^ than Mg^2+^ in any of these solvent environment. This behavior agrees with the work by Karatrantos,^45^ in which classical MD simulations showed that larger divalent cations than Mg^+2^ (such as Ca^+2^, Sr^+2^, Ba^+2^) experience a stronger interaction with anions which leads to a stronger cation–anion coordination and thus to a decreased anion diffusion coefficient with cation size. *Ab initio* orbital calculations by Seki^59^ showed that Mg^+2^ formed more stable structure than Ca^+2^ with PC and TFSI^−^, however only one PC or TFSI^−^ molecule was incorporated in the calculation. This enhanced ion–pair interaction with Ca^2+^ is a key factor contributing to its lower diffusion relative to Mg^2+^, as observed in Fig. 2. Furthermore, the RDF neighboring distances remain nearly unchanged with increasing temperature, as shown in Fig. S4,† indicating a robust structural arrangement over the temperature range studied. By increasing the charge scaling factor of the ions, both the cation–solvent and cation–anion electrostatic interaction would increase which would lead to stronger correlations in RDFs. In particular, the contact cation–solvent pairs will increase (1st RDF peak of the black lines in Fig. 3) and there will exist more contact cation–anion pairs.

**Fig. 4:**
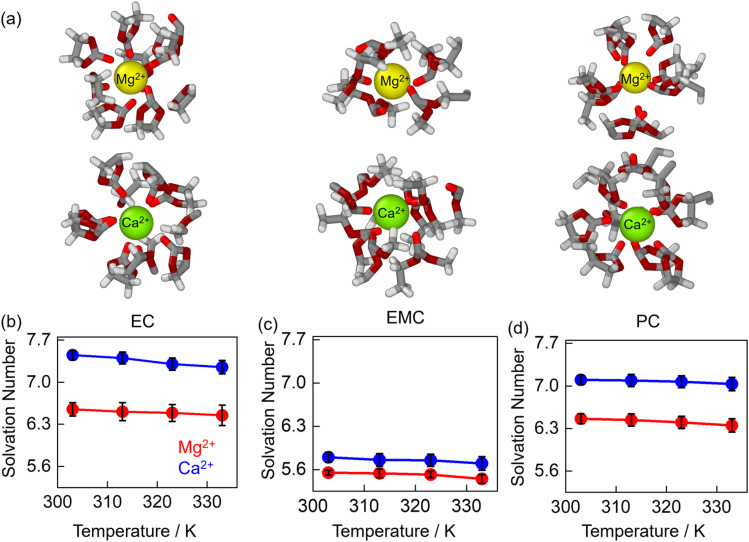
Simulated solvation structures of Mg^2+^ and Ca^2+^ with EC, EMC, and PC solvents (a), hydrogen, carbon, oxygen and carbonyl carbons are represented as white, grey, dark red and red color respectively. Average solvation numbers of Mg^2+^ (red) and Ca^2+^ (blue) as a function of temperature in EC (b), EMC (c), and PC (d) solvents.

Fig. 4(a) illustrates the simulated ball-and-stick representations of the solvation structures for Mg^2+^ (yellow) and Ca^2+^ (green) in EC, EMC, and PC solvents. The solvation numbers were determined using the first solvation shell cutoff obtained from the cation–solvent RDFs in Fig. 3. A strong interaction between the cations and solvents is evident, resulting in compact solvation shell formations. Ca^2+^ exhibits a denser solvation structure compared to Mg^2+^. Notably, the carbonyl carbon of these solvents is predominantly facing toward the cation in the solvation shells. The average solvation numbers for these cations across different temperatures are presented in Fig. 4(b–d) for EC, EMC, and PC solvents. The average solvation number remains relatively constant with temperature, highlighting the robust nature of these solvation shells. For Mg^2+^, the solvation numbers are approximately 6.6, 5.4, and 6.4 for EC, EMC, and PC, respectively, while for Ca^2+^, the values increase to 7.5, 5.9, and 7.1 for the same solvents. The linear geometry of EMC occupies more space within the solvation shell compared to the cyclic geometries of EC and PC, leading to comparatively lower solvation numbers in EMC. This structural distinction underscores the influence of solvent geometry on solvation dynamics and coordination environments.

### 3.3 Residence time

**Fig. 5:**
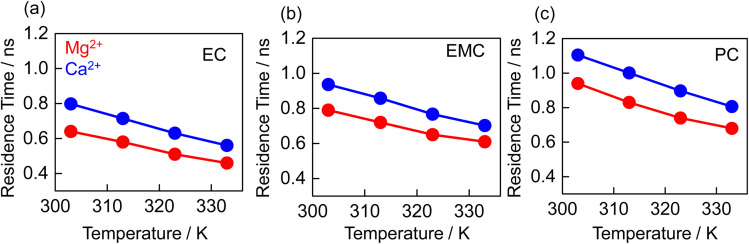
Simulated residence time of the Mg^2+^ (red), and Ca^2+^ (blue) in the solvation shell of EC (a), EMC (b), and PC (c).

The residence time (*R*_*ij*_(*t*)), representing the average duration that cations spend within the solvation shell of different solvents, is depicted in Fig. 5(a–c), which is calculated based on the exponential decaying of the solvent–cation autocorrelation function. Typically, it uses the autocorrelation function of a binary function *h*(*t*), which indicates whether a particle is in the specified cutoff region at a given time or otherwise.3
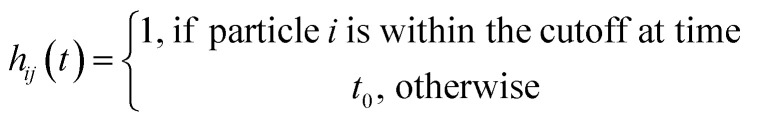


The autocorrelation function *h*(*t*) can be then calculated as

4 *R*_*ij*_(*t*) = <*h*_*ij*_(*t*)*h*_*ij*_(0)>

The time constant of an exact exponential was further determined by considering the point where the value of the autocorrelations function is 1/*e*.

The residence time exhibits a linear relationship with temperature. However, residence time is inversely proportional to the diffusion coefficient. Across the examined solvents and temperatures, the residence time for both Mg^2+^ and Ca^2+^ follows the trend PC > EMC > EC, which is the inverse of the observed diffusion trends. At lower temperatures, both Mg^2+^ and Ca^2+^ spend approximately 1.5 times longer in PC than in EC. The rigid solvation shells formed by PC enforce prolonged residence times for the cations, a phenomenon further supported by favorable ion–pair interactions. Comparing the two cations, Ca^2+^ consistently exhibits longer residence times than Mg^2+^ across all solvents, a trend opposite to their respective diffusion coefficients. This behavior is corroborated by the RDFs between cations and TFSI^−^, as shown in Fig. S4.† Ca^2+^ demonstrates a pronounced peak at 0.27 nm in all solvents, indicating stronger correlations with TFSI^−^ compared to Mg^2+^, which only shows a peak at 0.25 nm in PC. These findings suggest that the larger ionic size of Ca^2+^, coupled with its larger solvation shell and stronger ion–pair interactions, stabilizes Ca^2+^ within the solvation shell, thereby increasing residence time and reducing its diffusion coefficient.

## 4. Conclusion

This work explores the structure and dynamics of Mg^2+^ and Ca^2+^ in liquid electrolytes for the first time for EC and EMC solvents by means of molecular dynamics, emphasizing their relevance to energy storage systems. It was found that Mg^2+^ diffuses faster than Ca^2+^ across all tested solvents (EC, PC, EMC) due to its smaller ionic radius and weaker ion–pair interactions. Diffusion increases with temperature, with EC showing the highest diffusion rates among solvents. Solvent geometry impacts ion dynamics—cyclic solvents (EC and PC) enhance ion coordination, while linear solvents (EMC) reduce solvation due to spatial limitations. Mg^2+^ forms smaller, less compact solvation shells compared to Ca^2+^, which exhibits stronger ion–pair interactions and higher coordination numbers, particularly with TFSI^−^. Ca^2+^ has longer residence times in solvation shells, aligning with its lower diffusion rates, while Mg^2+^ exhibits shorter residence times due to its weaker interactions. The local coordination structures of both ions remain robust across the studied temperature range, indicating reliability under varying conditions. The study underscores the role of solvent selection and ion dynamics in optimizing divalent-ion battery performance, with Mg^2+^ emerging as a more favorable candidate for enhanced energy storage systems.

## Data availability

The data that support the findings of this study are included in this article and are available from the corresponding author upon reasonable request.

## Conflicts of interest

The authors declare no competing financial interests.

## Supplementary Material

RA-015-D5RA00757G-s001
