# Supplementary material for: Interplay between organic solvent geometry and divalent cation dynamics in divalent metal batteries
Source: RSC Adv. 2025 Apr 7;15(14):10851–60. doi: 10.1039/d5ra00757g (PMC11973965; doi:10.1039/d5ra00757g)
Supplement: RA-015-D5RA00757G-s001 [file RA-015-D5RA00757G-s001.pdf]

Supplementary Information (SI) for RSC Advances.
This journal is © The Royal Society of Chemistry 2025

Supporting Information for

Interplay Between Organic Solvent Geometry and Divalent Cation Dynamics

Nazifa Jahan Pranti[1], *Sharifa Faraezi*[1], *Tomonori Ohba*[2], *Argyrios V. Karatrantos,*[3] *and Md Sharif Khan*[1]*

[1]Center for Interdisciplinary Chemistry Research (CICR). Dhaka, Bangladesh

[2]Graduate School of Science, Chiba University, 1-33 Yayoi, Inage, Chiba 263-8522,Japan

[3]Luxemburg Institute of Science and Technology, 5, avenue des Hauts-Fourneaux,

L-4362 Esch-sur-Alzette, Luxembourg

Corresponding Author

* E-mail: sharifkhanjnu@gmail.com

Figures S1 –S5

	Atom Type	Sigma (nm)	Epsilon (kj / mol)
EC	OE	2.96000e-01	8.7864000e-01
	CE	3.75000e-01	4.393200e-01
	OSE	3.00000e-01	7.11280e-01
	C1E	3.50000e-01	2.7614400e-01
	C2E	3.50000e-01	2.7614400e-01
	H2E	2.42000e-01	6.276100e-02
	H1E	2.42000e-01	6.276100e-02
PC	OP	2.96000e-01	8.7864000e-01
	CP	3.75000e-01	4.393200e-01
	OAP	3.00000e-01	7.11280e-01
	C2P	3.50000e-01	2.7614400e-01
	C1P	3.50000e-01	2.7614400e-01
	OBP	3.00000e-01	7.11280e-01
	C3P	3.50000e-01	2.7614400e-01
	H1P	2.4200e-01	6.276000e-02
	H2P	2.42000e-01	6.276000e-02
	H3P	2.42000e-01	6.276000e-02
EMC	C1M	3.75000e-01	4.393200e-01
	O1M	3.00000e-01	7.11280e-01
	O2M	3.00000e-01	7.11280e-01
	O3M	2.96000e-01	8.7864000e-01
	C2M	3.50000e-01	2.7614400e-01
	C3M	3.50000e-01	2.7614400e-01
	C4M	3.50000e-01	2.7614400e-01
	H1M	2.42000e-01	6.276100e-02
	H2M	2.42000e-01	6.276100e-02
	H3M	2.42000e-01	6.276100e-02
	H4M	2.42000e-01	6.276100e-02
	H5M	2.42000e-01	6.276100e-02
	H6M	2.42000e-01	6.276100e-02
	H7M	2.42000e-01	6.276100e-02
	H8M	2.42000e-01	6.276100e-02
TFSI	NJ	3.25000e-01	7.1128e-01
	SJ	3.55000e-01	1.0460e-00
	OJ	2.96000e-01	8.7864e-01
	CJ	3.50000e-01	2.7614e-01
	FJ	2.95000e-01	2.2175e-01
Mg^{2+}	Mg	2.38500e-01	6.2000e-01
Ca^{2+}	Ca	2.70800e-01	12.200e-01

Table S1: Force field parameters of the different solvent and TFSI used in this study

	Solvent Number	Cation Number	Anion Number
**EC**	1128	37	74
**PC**	888	37	74
**EMC**	734	37	74

Table S2: Total number of solvents, cations and anions used in the simulation box

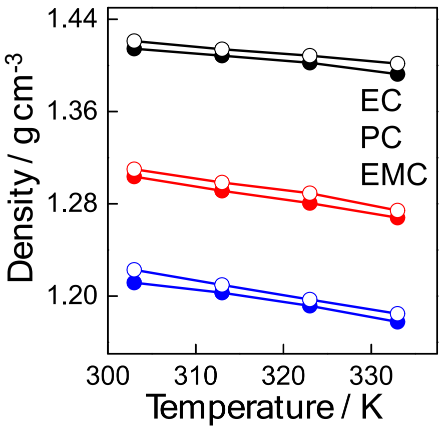

Figure S1: Change of the density of the electrolytes systems of Mg^{2+} (closed circle) and Ca^{2+} (open circle) as a function of temperature

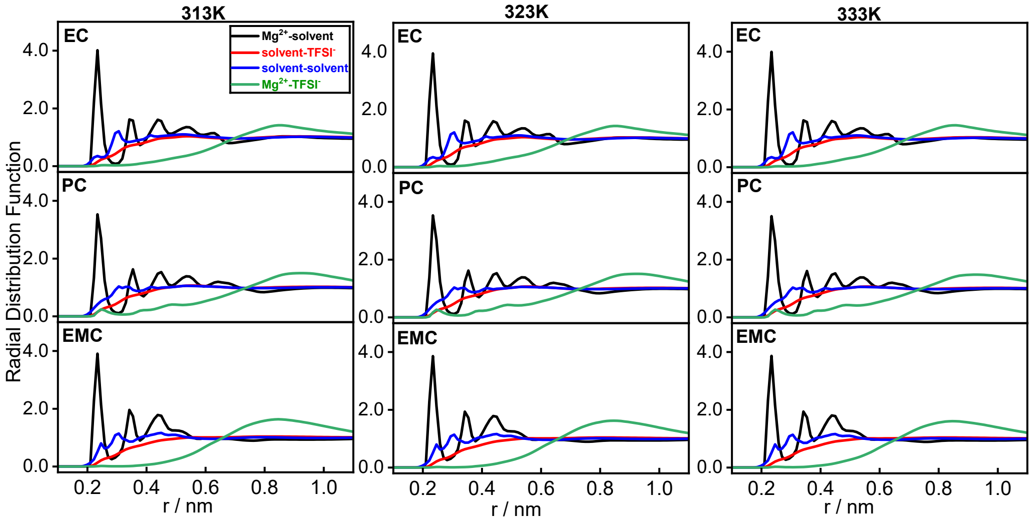

Figure S2: Radial distribution function of the different correlations Mg^{2+} - solvent (black), solvent – solvent (Blue), $TFSI^-$ - solvent (red), and Mg^{2+} - $TFSI^-$ (green) in different solvents at 313, 323 and 333 K temperatures.

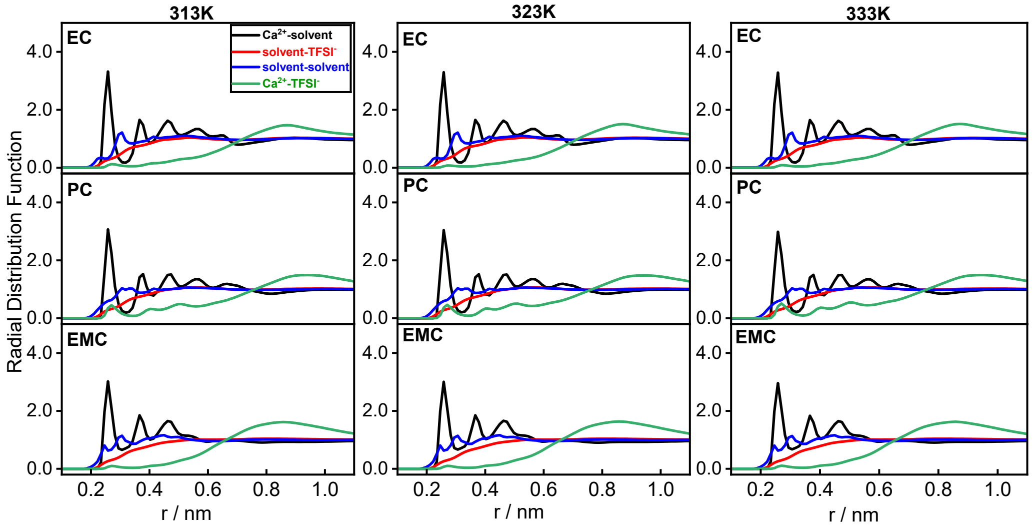

Figure S3: Radial distribution function of the different correlations Ca^{2+} - solvent (black), solvent – solvent (Blue), $TFSI^-$ - solvent (red), and Ca^{2+} - $TFSI^-$ (green) in different solvents at 313, 323 and 333 K temperatures.

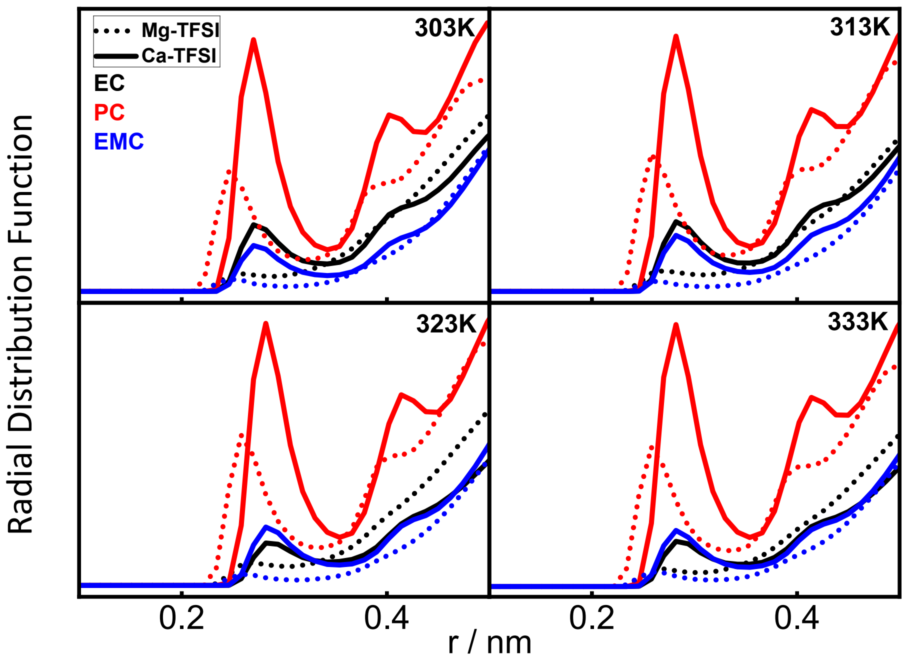

Figure S4: Radial distribution function between the Mg^{2+} - $TFSI^{-}$ and Ca^{2+} - $TFSI^{-}$ in different solvents at different applied temperatures.

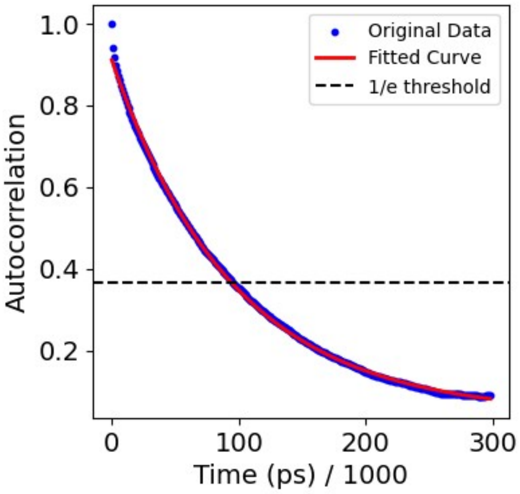

Figure S5: Example fit for the autocorrelation of the residence time to the equation of 4 of the main manuscript, here it is for Mg^{2+} ion in PC solvent.
